# Cellular Therapy in High-Risk Relapsed/Refractory Chronic Lymphocytic Leukemia and Richter Syndrome

**DOI:** 10.3389/fonc.2022.888109

**Published:** 2022-04-28

**Authors:** Maria Chiara Barbanti, Niamh Appleby, Murali Kesavan, Toby Andrew Eyre

**Affiliations:** ^1^ Department of Clinical Haematology, Oxford Cancer and Haematology Centre, Churchill Hospital, Oxford University Hospitals NHS Trust, Oxford, United Kingdom; ^2^ Clinical Trials Unit, Department of Oncology, Churchill Hospital, Oxford University Hospitals NHS Trust, University of Oxford, Oxford, United Kingdom

**Keywords:** CLL (chronic lymphocytic leukemia), CAR (chimeric antigen receptor) T-cell therapy, richter syndrome, cellular therapy, allogenic stem cell transplantation

## Abstract

Despite the development of highly effective, targeted inhibitors of B-cell proliferation and anti-apoptotic pathways in chronic lymphocytic leukemia (CLL), these treatments are not curative, and many patients will develop either intolerance or resistance to these treatments. Transformation of CLL to high-grade lymphoma—the so-called Richter syndrome (RS)—remains a highly chemoimmunotherapy-resistant disease, with the transformation occurring following targeted inhibitors for CLL treatment being particularly adverse. In light of this, cellular therapy in the form of allogenic stem cell transplantation and chimeric antigen receptor T-cell therapy continues to be explored in these entities. We reviewed the current literature assessing these treatment modalities in both high-risk CLL and RS. We also discussed their current limitations and place in treatment algorithms.

## Introduction

Substantial progress in understanding the pathobiology of chronic lymphocytic leukemia (CLL) has led to the development of drugs targeting key mechanisms of tumor proliferation and survival. Agents targeting the B-cell receptor (BCR) signaling cascade and B-cell lymphoma-2 (BCL2) expand treatment options for high-risk CLL including *TP53*-disrupted and relapsed/refractory (R/R) disease. While combination therapy can achieve deep and durable remissions, CLL remains incurable. High-grade transformation of CLL into aggressive B-cell lymphoma called Richter syndrome (RS) complicates CLL in 2%–15% (1–4). The wide range in incidence may be explained by the heterogeneous mutational status of CLL patients from different studies. In fact, specific biomarkers (e.g., NOTCH1, TP53 abnormalities, and trisomy 21) coupled with definite microenvironmental interactions associate to a higher risk of RS transformation (5, 6). Disease progression and high-grade transformation are a frequent cause of targeted therapy discontinuation in trial (3, 7) and non-trial (8–11) populations. Infrequently, RS presents *de novo* in untreated CLL patients.

Most RS cases represent transformation to a clonally related activated B-cell-type diffuse large B-cell lymphoma (DLBCL) (90%–95%), with a small proportion transforming to Hodgkin lymphoma (12). RS shares morphological characteristics with DLBCL, but its molecular profile is distinct. RS is enriched for mutations in poor-risk CLL drivers and the DNA damage response pathway (13).

Therapy for RS typically mirrors DLCBL, but outcomes are considerably worse (5, 14, 15) with median overall survival (OS) of 6–12 months (16–19). Intensification with hyper-CVXD (fractionated cyclophosphamide, vincristine, liposomal daunorubicin, dexamethasone with or without methotrexate) or OFAR (oxaliplatin, fludarabine, cytarabine, and rituximab) protocols may deliver improved responses, but responses are not sustained and OS remained <12 months (14, 20–23). Novel therapies targeting the BCR pathway continue to be explored in RS. Ibrutinib (24), acalabrutinib (25), or venetoclax monotherapy experience is reported in small series, with a short progression-free survival (PFS). Acalabrutinib plus R-CHOP is being examined in the STELLAR trial (26). Venetoclax with dose-adjusted R-EPOCH has shown promise, albeit in a selected cohort (27).

## History of Allogenic Stem Cell Transplantation in CLL

Given the evidence for graft-versus-leukemia (GVL) effect (28–31), there is continuing interest in defining the exact role of allogenic hematopoietic stem-cell transplantation (alloSCT) in CLL. Prospective data demonstrated a promising 2- to 6-year event-free survival (EFS) and OS rates ranging 30%–70% following reduced intensity conditioning (RIC) (32–34), demonstrating curative potential for R/R CLL patients. However, owing to significant inherent risks (33), alloSCT has historically been reserved for patients with sufficiently high-risk disease in the context of conventional chemoimmunotherapy induction.

Based on the 2007 EBMT consensus paper, high risk was defined in younger/fit patients as non-response or relapse within 24 months of having achieved a response to purine-analogue-based induction or post-autologous transplantation and the presence of deletion of 17p13 [del(17p)] by fluorescence *in situ* hybridization (FISH) or TP53 mutation by sequencing (28). Based primarily on retrospective data, guidelines advocated for the early consideration of related or unrelated donor alloSCT during CLL therapy in high-risk individuals (28). Complex karyotype (CK) defined as ≥3 distinct chromosomal abnormalities, in more than one metaphase, is increasingly recognized as heralding an adverse clinical course and informs selection of patients for cellular therapies (35–38).

In the pre-novel therapy era, these recommendations represented pragmatic guidance for the management of high-risk chemoimmunotherapy refractory patients and were accordingly widely adopted. However, the subsequent introduction and demonstration of long-term efficacy and safety of targeted inhibitors in CLL has unsurprisingly resulted in a marked reduction in transplantation (34, 39). The precise role of alloSCT within the current CLL treatment paradigm remains undefined.

## Outcomes in Dual Targeted (BTK and BCL2) Inhibitor-Exposed CLL Patients

With the advent of highly effective targeted inhibitors of Bruton tyrosine kinase (BTKi) (40–42) and BCL2 (43, 44) as treatment at frontline or relapse, the importance of adverse factors described in the immunochemotherapy era is challenged, e.g., 11q deletion and survival outcomes continue to markedly improve. Where access allows, most CLL patients will now cycle through time-limited venetoclax-based therapy (including potentially re-treatment) and continuous covalent BTK inhibition (cBTKi) with or without anti-CD20 monoclonal antibody. Accumulating evidence suggests that the order of such therapy is of relatively little importance with evidence of cross-resistance of drug classes lacking (45–47). Although high-risk patients are still often defined as those with IGVH-unmutated disease, TP53 mutations and/or 17p deletion, and CK, outcomes are demonstrably poor in the relatively small published patient series who develop resistance or intolerance to both major classes of targeted inhibitors, namely, cBTKi and BCL2i (11, 45, 48, 49).

A series of 17 patients who developed progressive disease (PD) after both cBTKi and BCL2i classes were recently reported (49). The cohort was heavily pre-treated with a median of four prior lines of therapy and displayed high-risk genomic features (CK in 12/12 tested, del17p/TP53 mutations in 15/17). Median time to progression on prior venetoclax was 24 months and that on prior cBTKi was 25 months. Progression following both agents was with CLL in 11 patients and RS in 6. Median OS at this juncture was only 3.6 months.

Phosphoinositide 3-kinase (PI3K) inhibition is a licensed option in this space; however, data are both limited and disappointing. Seventeen cBTKi/BCL2i-exposed patients observed an overall response rate (ORR) of 47% with a median PFS (mPFS) of only 5 months (45).

Non-covalent BTKis (ncBTKi) hold great promise in this dual-exposed space. Pirtobrutinib is a reversible ncBTKi active in C481S mutated and wild-type CLL (50, 51). Accumulating data from the BRUIN trial demonstrated an ORR of ~70% in 108 dual-exposed patients (median of five prior lines) and an mPFS of 18 months (52). Other ncBTKis such as MK1026 are in development and demonstrate efficacy in dual-exposed patient, but to date, data are less mature, and small patient numbers are reported (53). Despite clear promise, ncBTKis are not licensed to date.

The largest series describes outcomes in 125 “dual-exposed” CLL patients to cBTKi and venetoclax (54). Most common subsequent strategies included ncBTKi (n=45), cBTKi (n=43), immunochemotherapy (n=23), PI3Ki (n=24), alloSCT (n=17), chimeric antigen receptor (CAR) T-cell therapy (n=9), venetoclax re-treatment (n=6), and others (n=44). ORR and PFS estimates were as follows: CAR T-cell therapy (85.7%; mPFS, 4 months), alloSCT (76.5%; mPFS, 11 months), ncBTKi (75.0%; mPFS, not reached), PI3Ki (40.9%; mPFS, 5 months), CIT (31.8%; mPFS, 3 months), and venetoclax re-treatment (ORR, 40%; mPFS, 14 months), demonstrating the lack of clear standard approach in this setting.

In summary, this so-called “dual-exposed” patient cohort now represents the area of greatest and rapidly growing unmet medical need in CLL (54, 55).

## AlloSCT for CLL in the Targeted Inhibitor Era

Given the limited prognosis faced by multiply R/R patients in the current targeted inhibitor era, there is renewed interest in the curative potential of alloSCT. Several recent series highlight the efficacy of alloSCT in dual-exposed patients (**Table 1**).

**Table 1:** Allogenic stem cell transplantation for CLL and Richter syndrome.

Study group	Diagnosis	N.	OS	PFS	NRM
Kim et al. (56)	CLL	108	87% (3 years)	68% (3 years)	7% (3 years)
Roeker et al. (57)	CLL	65	81% (2 years)	63% (2 years)	13% (2 years)
Lahoud et al. (58)	CLL	35	66% (2 years)	46% (2 years)	26% (2 years)
Cwynarski et al. (59)	RS	25	36% (3 years)	27% (3 years)	26% (3 years)
Kim et al. (60)	RS	28	53% (4 years)	39% (4 years)	29% (4 years)
Lahoud et al. (58)	RS	23	74% (2 years)	65% (2 years)	13% (2 years)
Herrera et al. (61)	RS	118	52% (3 years)	43% (3 years)	27% (3 years)
Kharfan-Dabaja et al. (62)	RS	19	50% (4 years)	50% (4 years)	40% (4 years)

CLL, chronic lymphocytic leukemia; NRM, non-relapse mortality; OS, overall survival; PFS, progression-free survival; RS, Richter syndrome.

The Dana–Farber Cancer Institute (DFCI) reported outcomes of 108 RIC alloSCT for high-risk CLL, defined as any of the following: del(17p); ≥3 prior therapies; CK (≥3 abnormalities); IGHV unmutated; R/R to fludarabine, cyclophosphamide, and rituximab (FCR) prior to targeted therapy; poor response to prior chemoimmunotherapy; and poor response to targeted therapy. Thirty patients received prior targeted inhibitors, and 93% were refractory to ≥2 agents. The median age was 60 years, median prior therapies was 4, 76% had del(17p), 46.2% had ≥5 cytogenetic abnormalities, and 78.9% were IGHV unmutated. Median time to transplant from first-line therapy was 39 months. Remission status at alloSCT was CR in 20% and partial response (PR) in 73%. The 3-year OS and PFS were 87% and 69%, respectively. The cumulative incidence of relapse and non-relapse mortality (NRM) was 24% and 7%, respectively. The hematopoietic cell transplantation-specific comorbidity index (HCT-CI) was the only baseline clinical features (including HLA matching status, number and type of prior targeted inhibitors, and adverse genetic features) associated with an increased risk of death [hazard ratio (HR), 1.4; p=0.032] on univariable analysis (56).

The above data supported by a US/European collaboration where outcomes of 65 patients treated predominantly with RIC alloSCT following exposure to ≥1 targeted therapy are reported. Most patients had adverse genetic features including *TP53* mutation (51%), del(17p) (44%), and CK (50%). Two-year OS, PFS, NRM, and relapse incidence was 81%, 63%, 13%, and 27%, respectively. Grade ≥3 graft-versus-host disease (GVHD) developed in 27%, with a day+100 cumulative incidence of moderate–severe acute GVHD of 24%. Critically, adverse genetics features, prior number/type of targeted inhibitor exposure, remission status (CR vs. PR), and transplant characteristics were not independently associated with PFS/OS (57).

The most recent small (n=35) US series also analyzed the efficacy of RIC alloSCT in high-risk CLL patients (n=35), including a subset with RS. Of the CLL cohort without RS, 85% had adverse genetic features, and 65% were in PR at alloSCT. The 5-year PFS and OS was 40% and 58%, respectively. There was no statistically significant difference between RS and non-RS patients. Outcomes were again agnostic to adverse baseline genetic characteristics and prior targeted inhibitor exposure. The key clinical features associated with an improved PFS/OS following RIC alloSCT were treatment-sensitive response and ≤3 lines of prior therapy at alloSCT. Use of total body irradiation (TBI) containing RIC regimens was associated with an inferior PFS, OS, and relapse-free survival (58).

Taken together, these retrospective series highlight the efficacy and safety of RIC alloSCT in patients with high-risk R/R CLL following targeted inhibitor exposure and provide evidence for a durable graft-versus-leukemia effect. Critically, they advocate for the early identification of eligible patients and prioritization of alloSCT in those with treatment-sensitive disease.

Within the current CLL treatment paradigm, the curative potential of alloSCT must be balanced against novel agents accessible within trials and the well-described risks, which preclude a significant proportion of patients by virtue of their age, frailty, or comorbidities. For this subset of high-risk patients, alternative novel strategies, including cellular therapies and ncBTKi should be explored.

## Chimeric Antigen Receptor T cell Therapy in CLL

Over the past decade, CAR T-cell therapy has revolutionized the treatment of non-Hodgkin lymphoma (NHL). Three pivotal trials in multiply R/R-aggressive NHL patients demonstrated ORR rates of 52%–74% with 1-year OS rates of 48%–59% (63–65), resulting in the incorporation of this option in clinical practice (66–68). CAR T-cell therapy has been explored in CLL, first as monotherapy and recently in combination with ibrutinib (**Table 2**).

**Table 2:** Chimeric antigen receptor (CAR) T cell therapy in CLL and Richter syndrome.

Study group	Diagnosis	N.	ORR	CR	CRS	Neurotoxicity
Turtle et al. (69)	CLL	19	74%	11%	95%	37%
Siddiqi et al. (70)	CLL	23	82%	45%	74%	39%
Gauthier et al. (71)	CLL	19	83%	22% (all CRi)	74%	26%
Liu et al. (72)	CLL	5	80%	40%	0	0
Kittai et al. (73)	RS	9	89%	55%	100%	33%
Benjamini et al. (74)	RS	8	71%	71%	87%	37%
Turtle et al. (69)	RS	5	60%	40%	40%	20%
Ortiz-Maldonado et al. (75)	RS	5	80%	60%	80%	0

CLL, chronic lymphocytic leukemia; CR, complete response; CRi, complete response incomplete; CRS, cytokine release syndrome; ORR, overall response rate; RS, Richter syndrome.

Turtle and colleague enrolled 24 R/R CLL patients in a phase I/II trial where a defined composition of autologous CD4+- and CD8+ CD19-specific CAR T cells were infused following lymphodepletion. Eighty-three percent developed cytokine release syndrome (CRS), but only 25% (n=6) required tocilizumab and corticosteroids. Thirty-three percent had concomitant neurotoxicity, with 5/8 reaching grade 3 and one fatal event (69). These data are in line with relapsed/refractory DLBCL data, where CRS and neurotoxicity were reported in 42%–92% and 21%–67% of patients, respectively (63–65, 76). Notably, the ORR was 71% with an mPFS of 12.3 months (69).

The recent TRANSCEND CLL004 study enrolled 23 R/R CLL/SLL patients to receive Lisocabtagene maraleucel (Liso-cel). ORR and CR were achieved in 82% and 45%, respectively, with 75% and 65% of patients (n=20) achieving MRD negativity in peripheral blood and bone marrow, respectively. mPFS was 18 months but was significantly longer in those who achieved MRD negativity in blood and/or marrow. CRS complicated the course in 74% (9% grade 3), and 39% had neurological immune-related toxicity (22% grade 3–4) (70).

In both studies, patients had received at least two prior lines of therapy (100% had ibrutinib; 25%–65% had venetoclax), and the majority presented high-risk features including mutated TP53 and del(17p) (69, 70).

Recent data have demonstrated the persistence of CAR T cells at more than 10 years follow-up in two CLL patients who remained in complete remission. This study suggested the presence of distinct CAR T-cell populations possibly contributing to different phases of the anti-leukemia response. In one patient, an expansion of CD8+ or CD4−CD8− Helios^hi^ γδ CAR T cells in the first months after the infusion was seen, whereas later time points showed that a predominance of CD4+ CAR T cells was observed. In addition, both phenotype and antigenic signaling pathway analysis suggest that CAR T-cell proliferation was likely maintained through ongoing antigenic signaling through the transduced CAR (77).

Multiple groups are investigating strategies to improve CAR T-cell function in CLL patients. Several studies showed that ibrutinib is capable of modulating the immune dysfunction characterizing CLL patients by increasing Th1 and Th17 subsets (78, 79) and possibly reversing the exhausted T-cell phenotype associated to the expression of PD-1 and cytotoxic T-lymphocyte-associated protein 4 (CTLA-4) on lymphocytes (79, 80). Altogether, these findings suggest that BTKi could enhance CAR T-cell expansion and effector function.

An *in vitro* study from Fan and colleagues investigated the effect of ibrutinib on CLL patient-derived CAR T-cell production and found that the viability and expansion were increased. In addition, the CAR T-cell pool displayed a decreased expression of exhaustion markers (PD-1, TIM-3, and LAG-3) and was enriched with less-differentiated cells (81), which are thought to have the greatest capacity of engraftment and long-term persistence *in vivo* (82–84).

Following these promising data, a recent study on R/R CLL patients (n=19) investigated the effect of ibrutinib administered from 2 weeks before leukapheresis to 3 months after CAR T-cell infusion. The combined treatment showed good tolerability and efficacy with an ORR of 83% and 61% of patients achieved marrow MRD negativity by IGH sequencing. Ibrutinib appeared to mitigate the CRS severity despite equivalent CAR T-cell expansion (71).

The inhibition of PI3K signaling during manufacturing has been proposed as an alternative strategy to produce less differentiated and exhausted CAR T cells. Funk and colleagues showed that the *in vitro* addition of duvelisib (PI3Kδ/γ inhibitor) can decrease the expression of exhaustion markers; increase the number of T-stem cell memory, naive, and memory cells; and normalize the CD4/CD8 ratio (85).

Finally, Liu and colleagues conducted a phase 1/2 study using HLA-mismatched anti-CD19 CAR-NK cells derived from cord blood in 11 patients (5 CLL, 1 concomitant RS). Notably, no patients experienced CRS or neurotoxicity. At a median follow-up of 13.8 months, three CLL patients obtained a CR, which was maintained at last follow-up although with the use of post-remission therapy. Despite some limitations, this proof of concept may ultimately lead to the possibility of well-tolerated NK-based off-the-shelf product (72).

## AlloSCT for Richter Syndrome

While the outcomes for RS are dismal with conventional chemoimmunotherapy, the role of cellular therapy remains somewhat uncertain. Published data supporting alloSCT in RS are predominantly retrospective, single-center studies and report a selected RS cohort enriched for younger, fit, chemosensitive patients. Four of the 204 patients proceeded to alloSCT in one large single-institution publication of biopsy-proven RS (86), underlying the unmet need for effective induction therapies and the rarity of transplant-eligible RS patients.

EBMT reported on 25 RS patients who underwent alloSCT between 1997 and 2007 in the pre-targeted inhibitor era. One-third were chemo-refractory. The 3-year NRM was 26%, and OS was only 36%. The authors concluded that alloSCT is a viable therapeutic option for chemosensitive RS (59). Outcomes from the pre-BTKi era may not be applicable to contemporary practice.

Many recent reports are single center but describe similarly unsatisfactory outcomes in the targeted inhibitor era. One-year NRM ranges 24%–40% and 4-year OS at 49%–53% (60, 62). A systematic review and pooled meta-analysis of studies reporting ≥10 RS alloSCTs report an NRM of 24% and OS of 49% (87).

Two recent retrospective studies describe the experience of alloSCT for RS in the BCR inhibitor era. The 1-year NRM remains significant at 12%–23%. In both groups, one-third of the patients relapsed within 3–5 years (58, 61).

Comparable outcomes for RIC alloSCT for 35 R/R CLL and 23 RS were observed (58). All RS patients were considered for alloSCT at first remission. In univariate analysis, R/R CLL and treatment-responsive RS had comparable NRM, PFS, and OS following allograft.

Disease response status pre-alloSCT is predictive of outcome in the EBMT, Memorial Sloan Kettering and CIBMTR series (58, 59, 61). Thrombocytopenia, high LDH, and HCT-CI ≥2 identified patients at increased risk (60). While ≥3 lines of therapy were associated with adverse outcomes, there was no significant difference in outcomes between patients exposed and naive to BTKi, BCL2i, or PI3K inhibitors (58, 60, 61).

There remains a paucity of data to guide decisions regarding the source of stem cells, conditioning regimes, and GVHD prophylaxis. The MSK series observed inferior outcomes with TBI-containing conditioning and recommended against its use in this population. As most RS patients are older adults, non-myeloablative regimes using mobilized peripheral blood stem cells (PBSCs) are frequently employed.

Data on cellular therapy for Hodgkin-like transformation of CLL is even scarcer. One Hodgkin-like RS was reported in the Dana–Farber series; Hodgkin-like RS was excluded from the CIBMTR publication (60, 61).

The literature on alloSCT for RS is confounded by heterogeneous RS populations, variable approaches to alloSCT, and a rapidly changing therapeutic landscape for CLL and RS. In both high-risk CLL and RS, prior exposure to BTKi or BCL2i does not appear to confer an adverse prognosis in those receiving an alloSCT. Both NRM and relapse remain significant challenges in this population.

## Chimeric Antigen Receptor T Cell Therapy in Richter Syndrome

The promising results of CAR T-cell therapy in aggressive NHL prompted studies in RS. Data on CAR T-cells efficacy in this setting are limited and conflicting to date.

A recent retrospective study reviewed nine RS patients who were heavily pre-treated (median, four lines for CLL and/or RS). All patients had high-risk features including del(17p) (n=3), CK (n=6), and *TP53* mutation (n=2). Two patients received a BTKi as bridging before Axicabtagene ciloleucel (Axi-cel) infusion, while five other patients continued the BTKi for ≥30 days after the infusion. CRS occurred in all patients (grade ≤2, n=8; grade 4, n=1), whereas grade ≥3 neurotoxicity occurred in three patients. Five patients achieved CR, and three patients obtained a PR. One patient died of bacterial pneumonia. At a median follow-up of 6 months, only one patient had progressed, whereas all the others showed sustained responses (73).

Another cohort of eight patients with similar baseline characteristics was enrolled in a single-center phase 2 trial conducted in Israel exploring the use of CAR T cells after targeted therapies. At a median follow-up of 6 months, five patients achieved CR, while three patients progressed. Seven patients developed CRS (grade 3–4, n=3) and three developed neurotoxicity (grade 3, n=2) (74).

Heterogeneous responses were observed in the five RS patients enrolled in the study conducted by Turtle and colleagues. After CAR T-cell product JCAR017 infusion, CR was observed in two patients, PR in one patient, and PD in two other cases (69).

Interestingly, a Spanish phase I study infused ARI-0001, a novel CAR T-cell construct, to five RS patients using a fractionated dose scheme. The CRS rate was 80%, whereas neurotoxicity was not observed. One patient received only 10%–40% of the expected cell dose due to CRS. Four patients responded to treatment (CR, n=3), while one remained with SD according to iwCLL/Lugano criteria. However, MRD negativity was achieved in all patients both in peripheral blood and bone marrow (75). ARI-0001 has recently been approved by the Spanish Medicines Agency (AEMPS) for patients with R/R acute lymphoblastic leukemia (ALL) >25 years of age.

Other small studies suggested lack of response to CAR T-cell therapy or non-sustained response in the context of RS (88, 89).

Published reports examining the role of CAR T-cell therapy for RS are limited by small numbers, variable approaches to concurrent therapies, and short follow-up. Despite these restrictions, disease responses are observed at least for a minority. However, the above reports highlight manageable toxicity and promising outcomes for heavily pre-treated and high-risk patients with few therapeutic options left. Further work is needed to determine the precise role of CAR T-cell therapy in the treatment of RS.

## Discussion

High-risk R/R CLL—particularly patients now “dual exposed” to BTKi and BCL2i—and R/R RS remain areas of ongoing clinical need and investigation. Cellular therapy in the form of alloSCT represents an ongoing option for fit, younger CLL patients achieving disease control in these settings and has demonstrable utility in the targeted inhibitor era. While CAR T-cell therapy provides cause for optimism, the clinical data supporting this therapeutic modality at present are limited. Ongoing investigation into improving T-cell function and further prospective clinical data are needed before this treatment becomes a *de facto* standard of care approach across a wider range of R/R CLL and RS patients. Despite this, the limited data in R/R RS are promising, and, where available, this modality could be considered in R/R RS patients who can be bridged to reinfusion with reasonable performance status and disease control.

## Author Contributions

All authors listed have made a substantial, direct, and intellectual contribution to the work and approved it for publication.

## Funding

This study was supported in part by the Oxford Biomedical Research Centre.

## Conflict of Interest

NA received speakers fees from Gilead and research funding from Janssen. TE received education honorarium, advisory board honorarium, and travel support from Roche; received honorarium, research support, and travel to scientific conferences from Gilead; received advisory board honorarium from KITE; received honorarium from Janssen; received honorarium and travel to scientific conferences from Abbvie; received honorarium and research funding from AstraZeneca; received advisory board honorarium and a member of trial steering committee from Loxo Oncology; received advisory board honorarium and research funding from Beigene; advisory board honorarium from Incyte; and received Advisory Board Honorarium Secura Bio.

The remaining authors declare that the research was conducted in the absence of any commercial or financial relationships that could be construed as a potential conflict of interest.

The handling editor JR declared a past co-authorship with the author TE.

## Publisher’s Note

All claims expressed in this article are solely those of the authors and do not necessarily represent those of their affiliated organizations, or those of the publisher, the editors and the reviewers. Any product that may be evaluated in this article, or claim that may be made by its manufacturer, is not guaranteed or endorsed by the publisher.
